# Comorbidities, Clinical Presentation, Subtypes, and Treatment of HS Patients in Lithuania

**DOI:** 10.3390/jcm13133900

**Published:** 2024-07-03

**Authors:** Tadas Raudonis, Austėja Šakaitytė, Tomas Petras Vileikis, Vitalij Černel, Rūta Gancevičienė, Christos C. Zouboulis

**Affiliations:** 1 Clinic of Infectious Disease and Dermatovenereology, Institute of Clinical Medicine, Faculty of Medicine, Vilnius University, M. K. Čiurlionio g. 21, 03101 Vilnius, Lithuania; tomas.vileikis97@gmail.com (T.P.V.); ruta.ganceviciene@gmail.com (R.G.); 2 European Hidradenitis Suppurativa Foundation e.V., 06847 Dessau, Germany; christos.zouboulis@t-online.de; 3 Faculty of Medicine, Vilnius University, M. K. Čiurlionio g. 21, 03101 Vilnius, Lithuania; austeja.sakaityte@gmail.com (A.Š.); vitalijus@bardai.lt (V.Č.); 4 Departments of Dermatology, Venereology, Allergology and Immunology, Dessau Medical Center, Brandenburg Medical School Theodor Fontane and Faculty of Health Sciences Brandenburg, 06847 Dessau, Germany

**Keywords:** hidradenitis suppurativa, comorbidities, IHS4, DLQI

## Abstract

**Background**: Hidradenitis suppurativa (HS) is a persistent, recurring skin inflammatory condition linked to various comorbidities. Management involves antibiotics, hormone therapy, immune-modulating drugs, surgery, and treatment of comorbidities. The objectives of the study were to assess the comorbidities, clinical presentation subtypes, and applied treatment of patients with HS. **Methods**: Patients with HS who visited the Centre of Dermatovenereology at Vilnius University Hospital Santaros Klinikos in Lithuania underwent evaluation based on the guidelines of the European Hidradenitis Suppurativa Foundation Registry questionnaire. **Results**: The study included 49 patients, and 61.22% (*n* = 30) had comorbidities. A strong positive correlation was found between a family history of inflammatory diseases (69.38% (*n* = 34)) and the severity of HS according to Hurley stage (r = 0.71 *p* < 0.05). A statistically significant correlation (r = 0.944, *p* = 0.02) was found between metabolic comorbidities and Hurley stage. Patients on biologic treatment had a mean IHS4 of 7.38 at the beginning of treatment and 3.22 at follow-up (*p* < 0.05). For patients not on biologics, the initial IHS4 score was 6.21 and 5.42 at follow-up (*p* > 0.05). **Conclusions**: A family history of inflammatory diseases and metabolic comorbidities showed a strong correlation with HS severity. Treatment with biologics showed significant improvement in HS scores compared to systemic antibiotics.

## 1. Introduction

Hidradenitis suppurativa (HS), also known as acne inversa, is a lifelong inflammatory skin disease characterized by the presence of deep-seated nodules, abscesses, draining tunnels, and fibrotic scars. These distressing lesions primarily manifest in areas where skin folds meet and where apocrine glands are abundant. The most commonly affected regions include the armpits, groin, perianal region, perineum, and inframammary area [1]. The heterogeneous nature of HS has led to the identification of various stages and phenotypes within the disease spectrum [2].

HS, being a chronic inflammatory disease, exemplifies the connection between skin-related conditions and comorbid systemic diseases, as they share common inflammatory pathways. Patients with HS tend to have a higher burden of comorbidities compared to the general population, and HS itself is independently associated with several specific comorbid conditions (such as polycystic ovary syndrome, diabetes mellitus, metabolic syndrome, inflammatory bowel disease, psychiatric disorders, lymphoma, etc.) [3,4].

The initial pedigree studies revealed that familial occurrence was reported by only 30 to 40% of individuals with HS, indicating a limited influence of genetics as a risk factor in the development of the disease [5]. Nonetheless, recent twin studies have shown a significant heritability rate in HS, ranging from 77% to 80%, suggesting that genetic factors may have a more substantial impact on the condition than previously believed [6,7]. With the growing recognition of the role of genetics, it is crucial to conduct large-scale studies to gain a better understanding of the genetic architecture underlying HS.

The management of HS depends on the severity of the condition and may involve a combination of topical and systemic antibiotics, hormone therapy, immune-modulating medications, and surgical interventions [1]. Surgically addressing locally recurring lesions is a suitable approach, while medical treatment, either as a standalone therapy or in conjunction with surgery, is more appropriate for widespread lesions [8].

The aim of this study was to assess the comorbidities of patients with HS and differences in clinical presentation, subtypes, and treatment.

## 2. Materials and Methods

Patients with HS who visited the Centre of Dermatovenereology at Vilnius University Hospital Santaros Klinikos in Lithuania between March 2021 and June 2023 underwent evaluation based on the guidelines of the European Hidradenitis Suppurativa Foundation (EHSF) Registry questionnaire at different points in their disease and treatment progression. Comorbidities, family history of inflammatory diseases, lesion location, and clinical subtypes of HS using the Canoui-Pouitrine et al. [9] criteria were determined. Information about the previous and current treatment was documented. The assessment was conducted on changes in the dermatology life quality index (DLQI) and International Hidradenitis Suppurativa Severity Score System (IHS4) before and after treatment. Data were analyzed using MS Excel 2021 and IBM SPSS 26.0. The normal distribution of data was examined with the Shapiro–Wilk test, and the groups’ homogeneity was assessed using the chi-square test. Statistical significance was acknowledged when the value fell below the threshold of 0.05 (*p* < 0.05).

This research received approval from the Vilnius Regional Biomedical Research Ethics Committee (ethical approval number 2021/2-1310-793). All participants provided written informed consent prior to their involvement in the study.

## 3. Results

### 3.1. Demographics and Comorbidities

The study included 49 patients, and 57.14% (*N* = 28) of them were male. The average age of the subjects was 39.91 ± 13.665 years, average BMI was 28.44 ± 6.142, 30.61% (*N* = 15) were overweight, and 36.73% (*N* = 18) were obese; there was no statistical correlation between gender and BMI variables (Table 1).

It was found that 34.69% (*N* = 17) of subjects had severe acne, and in 64.70% (*N* = 11) of them, it was still ongoing; however, no correlation between acne and Hurley stage (r = 0.088) or severity of HS (r = 0.091) was found. We found that 61.22% (*N* = 30) of patients had comorbidities such as psoriasis, inflammatory bowel disease, joint, metabolic disease, dyslipidemia, and hypertension (Table 1).

**Figure 1:**
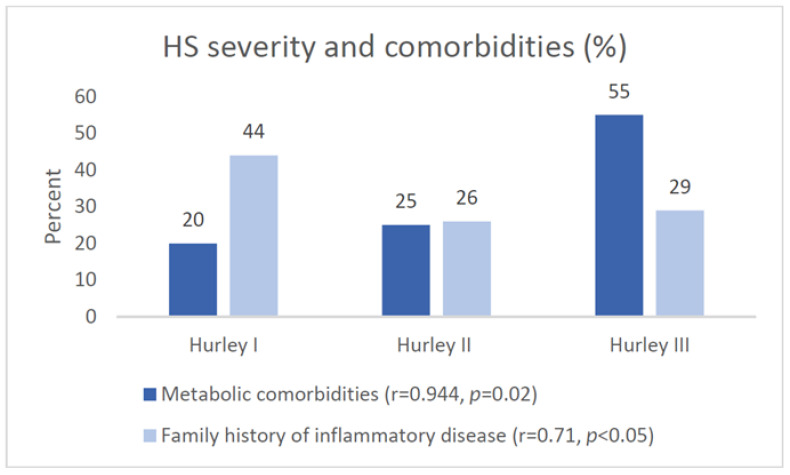
Association between Hurley stage and metabolic comorbidities, as well as family history, of inflammatory disease.

Only 18.36% (*N* = 9) had a family history of HS; however, a moderate positive correlation was found between a family history of inflammatory diseases (69.38% (*N* = 34)), which include acne, psoriasis, inflammatory bowel disease, and joint disease, and the severity of HS according to Hurley stage (r = 0.71, *p* < 0.05). We found that 30.6% (*N* = 15) of patients had comorbidities related to cardiovascular disease, and 60% (*N* = 9) of them had a positive family history of inflammatory diseases. Comparing the severity of HS, a strong statistically significant correlation (r = 0.944, *p* = 0.02) was found between metabolic comorbidities and Hurley stage, and 55.0% (*N* = 10) of them were Hurley stage III (Figure 1).

### 3.2. Clinical Presentation

We found that 75.51% (*N* = 37) had lesions in the axillary region, 59.18% (*N* = 29) in the groin area, 28.57% (*N* = 14) in the pubic, and 26.53% (*N* = 13) in other areas (Table 2). There were no significant differences between males and females. In 88% (*N* = 16) of obese subjects, the groin area was affected.

### 3.3. Clinical Subtypes of HS

The HS phenotype of all patients was determined using the Canoui-Pouitrine et al. criteria [9]. We found that 40.81% (*N* = 20) had an axillary mammary phenotype, 34.69% (*N* = 17) follicular, and 24.48% (*N* = 12) gluteal phenotype. A follicular phenotype had a higher percentage of non-smokers compared to the axillary mammary phenotype and more Hurley III subjects than the gluteal and axillary mammary phenotypes (Table 3).

### 3.4. Treatment

Before the assessment, patients used a variety of medications for HS, which are presented in Table 4.

After the initial assessment, the following medications were usually prescribed, which are presented in Table 5.

**Figure 2:**
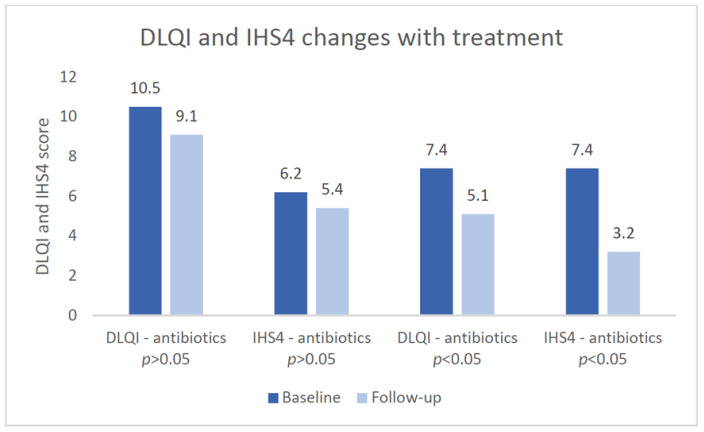
Systemic HS treatment impact on the DLQI and IHS4 scores.

We found out that 32.65% (*N* = 16) were on biological therapy, 65.30% (*N* = 32) were on long-term systemic antibiotics, 14.89% (*N* = 7) on both biologics and systemic antibiotics, and 44.68% (*N* = 21) were treated only with topicals. Patients on biologics initially had a mean DLQI of 7.4, systemic antibiotics of 10.5, and others of 7. Data from the follow-up were included after 6 months of the initial assessment and medication prescription. Patients on biologic treatment had a mean IHS4 of 7.38 at the beginning of treatment and 3.22 at follow-up (*p* < 0.05). Meanwhile, for patients not on biologics, the initial IHS4 score was 6.21 and 5.42 at follow-up (*p* > 0.05) (Figure 2). We found that 29.78% (*n* = 14) had recurrent inflammatory lesions in the last 4 weeks, with no differences between patients on biologics or systemic antibiotics.

## 4. Discussion

### 4.1. Comorbidities

Patients with HS experience an exceptionally elevated burden of comorbidities [9,10]. Dyslipidemia, hypertension, obesity, thyroid disorders, psychiatric disorders, arthropathies, and polycystic ovarian syndrome have each shown independent associations with HS [9]. Although the precise relationship between HS and its comorbidities is not fully understood, numerous related conditions are characterized by inflammation as well [11]. Regardless of age, gender, socioeconomic status, smoking, and medication use, patients with HS experience a notable rise in major adverse cardiovascular events, such as ischemic stroke, myocardial infarction, and cardiovascular-related death, as well as overall mortality. Elevated levels of C-reactive protein and tumor necrosis factor-α, which have been linked to atherosclerosis, are also heightened in HS [12]. The connection between chronic inflammation and HS is observed in association with metabolic syndrome, a condition that comprises diabetes mellitus, dyslipidemia, hypertension, and obesity [13,14,15,16]. For both hospitalized and nonhospitalized individuals with HS, the odds ratios for being diagnosed with metabolic syndrome, as compared to healthy patients, are 3.89 and 2.08, respectively [16]. In our study, a similar tendency was observed: 30.6% of the patients had comorbidities associated with cardiovascular disease, and among them, 60% had a positive family history of inflammatory diseases.

An association also exists between HS and psoriasis. An examination involving 68,836 psoriasis patients revealed a higher prevalence of HS in comparison to sex-, age-, and ethnicity-matched control subjects (0.3% versus 0.2%). Psoriasis patients who have concurrent HS tend to be younger and exhibit a greater prevalence of obesity and smoking [17].

Moreover, HS has been linked to various other conditions characterized by a proinflammatory state. Inflammatory bowel disease (IBD), notably Crohn’s disease (CD), exhibits potential epidemiological and pathogenic correlations with HS [18,19]. The analysis of four studies indicated that the prevalence of HS among patients with IBD and CD was found to be 12.8% and 17.3%, respectively. Moreover, HS populations exhibit a higher prevalence of inflammatory arthritis compared to the general population [10,20]. In a prospective study involving 640 patients, 3.7% exhibited concurrent spondylarthritis as a comorbidity, with HS preceding articular symptoms in over 90% of those individuals [20]. Consistent with the findings in the literature, our study showed that comorbidities such as psoriasis, inflammatory bowel disease, joint disorders, metabolic disorders, dyslipidemia, and hypertension were present in 61.22% of the patients. When assessing the severity of HS, a statistically significant correlation was identified between metabolic comorbidities and the Hurley stage, with 55.0% of those comorbid cases classified as Hurley stage III. This tendency was also outlined in the study by Kimball et al., which asserted that the higher the severity of the disease, the greater the burden of comorbidities, particularly those such as a sebaceous cyst, pilonidal cyst, kidney disease, hypertension, diabetes, deficiency anemia, and congestive heart failure [21].

Certain dermatological conditions display a shared cutaneous pathology with HS. Follicular occlusion, hyperplasia of the pilosebaceous apparatus, and bacterial invasion are contributing factors in acne conglobate, dissecting cellulitis, as well as HS. Collectively, these conditions form the follicular occlusion triad [22]. Inclusion of the pilonidal cyst completes the follicular occlusion tetrad [23]. Acne and HS can also be elements of autoinflammatory syndromes. The presence of acne, pyoderma gangrenosum, and hidradenitis suppurativa (PASH) together defines an established syndrome. In cases where a patient has PASH along with the inclusion of pyogenic arthritis, the condition is labeled as PAPASH [24]. Ultimately, persistent HS has the potential to progress into squamous cell carcinoma. Approximately 4.6% of HS cases are linked to the presence of squamous cell carcinoma [25]. A link to dermatological conditions has been observed in our study as well. Severe acne was evident in 34.69% of the participants, and among them, it remains ongoing in 64.70% of cases. These numbers in the general HS population could be even higher, considering that the prevalence of acne is higher among females than males (similar to the higher prevalence of HS in the Western population), yet our study had a higher proportion of males than females [26,27].

### 4.2. Clinical Presentation and Subtypes of HS

According to the adapted Dessau definition, three diagnostic criteria need to be fulfilled: the existence of characteristic lesions, occurrence in typical areas, and chronicity [8]. Characteristic lesions encompass deeply situated, painful nodules, suppurative sinus tracts or channels, abscesses, and connected scars, as well as double- and multi-ended comedones, often referred to as “tombstone comedones” [28]. Abscesses and subcutaneous nodules have the potential to rupture, leading to bleeding and the release of a purulent discharge. This ongoing progression ultimately culminates in dermal contraction and fibrosis of the affected skin. The axillary, infra-, and inframammary; inguinal; perineal; and gluteal regions are frequently impacted anatomical areas [29]. Less commonly mentioned sites include the lower abdomen, suprapubic area, retroauricular region, eyelids, nape, and scalp [30,31,32]. A defining characteristic of HS is its chronic nature, marked by two recurrences within a 6-month period. These three criteria by themselves generally exhibit a high level of diagnostic sensitivity and specificity [33]. Our research showed that, in terms of lesion distribution, 75.51% were situated in the axillary region, 59.18% in the groin area, 28.57% in the pubic area, and 26.53% in other areas. No significant gender-based differences were observed. Among the obese participants, 88% exhibited involvement in the groin area.

The majority of patients present with more than one lesion during the time of diagnosis [34]. These lesions typically bring about sensations of discomfort, itching, and pain [35,36]. A significant number of patients undergo prodromal pain symptoms [37]. Various factors such as heat, sweating, physical exertion, shaving, and friction intensify symptoms [16,38]. The standard pattern involves acute flare-ups followed by phases of dormancy [37].

Various phenotypes of HS exist. The research conducted by Canoui-Poitrine et al. identified the presence of three distinct phenotypes: “axillary mammary”, “follicular”, and “gluteal”, with the majority (48%) having the “axillary mammary” phenotype [39]. In our research, 40.81% of participants displayed the “axillary mammary” phenotype, which exhibited a high prevalence of armpit and breast involvement, as well as hypertrophic scars. The remaining two phenotypes, labeled as “follicular” and “gluteal”, represent atypical variations of the condition [39,40]. In our study, the “follicular” phenotype, characterized by the presence of follicular lesions, including epidermal cysts, pilonidal sinus, and comedones, along with severe acne, was found in 34.69% of cases. The “follicular” phenotype showed a greater proportion of non-smokers in comparison to the “axillary mammary” phenotype, while in Canoui-Poitrine et al.’s study, the “follicular” phenotype was identified in 26% of patients, with a higher proportion of male participants and individuals who were current or former smokers [39]. In our investigation, this phenotype also had a higher number of Hurley III subjects than the “gluteal” and “axillary mammary” phenotypes. The Canoui-Poitrine et al. study also confirmed that patients with the “follicular” phenotype experienced more severe symptoms, earlier onset of the condition, and a longer duration of illness compared to the typical “axillary mammary” phenotype [39]. In our research, 24.48% of patients showcased the gluteal phenotype, which is marked by engagement with the gluteal region, the presence of follicular papules, and the development of folliculitis. According to Canoui-Poitrine et al., this phenotype had a higher proportion of smokers, a lower average BMI, and, surprisingly, less severe symptoms, despite a longer duration of HS compared to the “axillary mammary” phenotype [39].

### 4.3. Treatment

For individuals in Hurley stages I/II who have mild and localized HS marked by a small number of lesions, the use of topical clindamycin 1% is recommended when there are no deep-seated inflammatory lesions present [41]. The majority of our subjects were prescribed topical clindamycin with benzoyl peroxide but with limited efficacy. The use of intralesional steroids can be beneficial for managing acute inflammatory nodules in conjunction with other therapies across all stages of Hurley classification [42], and more than 20% of our subjects were previously treated with intralesional triamcinolone at 10 mg/mL, which helps to promptly reduce inflammation in HS lesions. For patients classified under Hurley stages I/II with multiple lesions and frequent worsening of symptoms, systemic tetracyclines can be used for 10–12 weeks [43,44]. For patients in Hurley stages II/III who have multiple ongoing lesions, the recommended treatment involves systemic clindamycin with rifampicin 300 mg twice daily for 10 weeks [43,44,45,46]. In our research, 65.30% of participants were undergoing long-term systemic antibiotic treatment with doxycycline or clindamycin/rifampicin. Patients on systemic antibiotics showed a mean baseline DLQI of 10.5, decreasing to 9.2 at follow-up, with initial and follow-up IHS4 scores of 6.21 and 5.42, respectively. In cases of moderate-to-severe HS where conventional treatments have proven ineffective, adalimumab should be regarded as the first choice among biologic agents [47,48,49]. The use of adalimumab for HS was evaluated in the PIONEER I and II trials. The clinical response rates at week 12 were notably greater in the groups receiving weekly adalimumab compared to the placebo. Specifically, in PIONEER I, it was 41.8% versus 26.0%, and in PIONEER II, it was 58.9% versus 27.6% [49]. In our study, 22.44% of the patients were treated with adalimumab. The patients on biologics had a mean DLQI of 7.4 at baseline and 5.1 at follow-up. Regarding the initial IHS4 score, the patients on biologic treatment had a mean score of 7.38, which decreased to 3.22 at follow-up. However, 29.78% of participants experienced lesion recurrence in the last 4 weeks, with no significant differences observed between those on biologics and those on systemic antibiotics.

Infliximab has demonstrated its effectiveness and could be taken into consideration as a secondary biologic option for individuals with moderate-to-severe HS; however, it still does not have an official indication for HS [50], and its efficacy is usually of limited duration due to the neutralizing antibody formation [51,52].

Translational research has revealed that IL-17A plays a pivotal role in HS [53]. Initially, case reports and open-label studies published after 2018 demonstrated clinical enhancements in patients treated with secukinumab. Two identical (SUNSHINE and SUNRISE), multicenter, randomized, placebo-controlled, double-blinded phase 3 trials were performed. In both trials, secukinumab demonstrated a clinical response rate of 42–46%, with better efficacy observed with biweekly administration, compared to 31–34% in the placebo group [54]. In our study, five patients were administered secukinumab treatment, but the investigation into its efficacy was not pursued due to the limited number of subjects.

### 4.4. Future Treatment Developments

IL-36 has been associated with the development of inflammatory bowel disease, psoriasis, acne, and HS. In these conditions, IL-36 has been linked with inflammation, the attraction of neutrophils, and the thickening of the epidermis. Spesolimab is presently employed to target IL-36. The approach of using spesolimab to target IL-36 for treating HS is well suited to the pathogenic framework of the disease. At present, the supporting evidence is primarily derived from individual cases and case series, but there are several studies underway to further investigate its potential efficacy [55].

The involvement of the Janus kinase (JAK)/signal transducer and activator of transcription signaling (STAT) has been linked to the pathophysiological processes of HS. Based on observations from skin biopsies and blood samples of individuals with moderate-to-severe HS, an 8-week treatment with the JAK1 inhibitor, povorcitinib, was associated with the reversal of a previously identified HS transcriptomic pattern. Additionally, there were dose-dependent alterations in several circulating proteins that may play a role in the development of the disease. These observed changes in biomarkers largely align with the mechanism of action of povorcitinib, which primarily affects the pathways controlled by JAK/STAT signaling. Furthermore, some biomarker changes appear to mirror the clinical effectiveness of the treatment, underscoring the potential for JAK1 inhibition to influence the fundamental disease processes in HS [56].

### 4.5. Surgery

A range of surgical interventions are at one’s disposal, and there is not a single optimal treatment, necessitating a personalized strategy for every patient [57].

Incision and drainage should not be seen as a standalone treatment option, as recurrence is nearly certain [58,59,60,61]. Surgical interventions, including limited excision, deroofing, and Skin-Tissue-sparing Excision with Electrosurgical Peeling (STEEP), can be employed for isolated lesions. In Hurley stage III, a comprehensive excision of the entire affected region, involving the removal of both inflamed and non-inflamed sinuses, nodules, and scar tissue, may be conducted as a preventative measure against recurrence [62,63,64]. The use of biologic therapy before and after surgery could result in a decreased likelihood of recurrence and a longer period free from the disease [65,66].

## 5. Conclusions

This study identified a positive correlation between a family history of inflammatory diseases and the severity of HS according to the Hurley stage. Metabolic comorbidities showed a strong correlation with HS severity, particularly in Hurley stage III cases. Most patients displayed lesions in the axillary region, but a substantial proportion also exhibited them in the groin area, with no notable gender differences. Patients on biologics showed a significant improvement in IHS4 scores. There were no significant differences in the recurrence of inflammatory lesions between patients on biologics or antibiotics.

## Figures and Tables

**Table 1:** HS patient demographic data and comorbidities.

Variables	Patients (*n* = 49)	*p*-Value
Age, years		
Mean ± SD	39.91 ± 13.665	0.565
Median (range)	39.24 (18.73)	
Sex		
Female, *n*, (%)	21 (42.85%)	0.378
Male, *n*, (%)	28 (57.14%)	
Average BMI kg/m^2^	28.44 ±6.142	
Ethnic origin:		
Caucasians	49 (100%)	
Smoking status:		
Smoker	18 (36.73%)	
Previous smoker	11 (22.44%)	
Non-smoker	20 (40.81%)	
Metabolic comorbidities:		
Hypertension	10 (29.4%)	
Dyslipidaemia	6 (12.24%)	
Diabetes	3 (6.12%)	
Metabolic disease	1 (2.04%)	
Inflammatory diseases:		
Acne	17 (34.69%)	
Pilonidal cyst	10 (29.4%)	
Psoriasis	4 (8.16%)	
Inflammatory bowel disease	3 (6.12%)	
Other comorbidities:		
Depression	8 (16.32%)	
Joint pain	6 (12.24%)	
Hurley stages:		
I	25 (51.02%)	
II	12 (24.48%)	
III	12 (24.48%)	

**Table 2:** Distribution of HS lesions.

Location of Lesions	All Patients (*N* = 49)	Males (*N* = 28)	Females (*N* = 21)	*p*-Value
Axillary	37 (75.51%)	20 (71.42%)	17 (80.95%)	0.295
Groin	29 (59.18%)	18 (64.28%)	11 (52.38%)	0.042
Pubic	14 (28.57%)	8 (28.57%)	6 (28.57%)	0.395
Other areas	13 (26.53%)	7 (25.00%)	6 (28.57%)	0.467
HS lesion types				
Axillary region:				
Abscesses	7 (18.91%)	4 (20%)	3 (17.64%)	0.271
Nodules	21 (56.75%)	9 (45%)	12 (70.58%)	0.249
Fistulas	9 (24.32%)	5 (25%)	4 (23.52%)	0.543
Groin region:				
Abscesses	7 (24.13%)	5 (27.77%)	3 (27.27%)	0.042
Nodules	15 (51.72%)	9 (50%)	7 (63.63%)	0.284
Fistulas	7 (24.13%)	4(22.22%)	1 (9.09%)	0.031
Pubic region:				
Abscesses	4 (28.57%)	2 (25%)	1 (16.66%)	0.038
Nodules	8 (57.14%)	4 (50%)	4 (66.66%)	0.782
Fistulas	2 (14.28%)	2 (25%)	1 (16.66%)	0.081
Other regions (face, chin, abdomen, breasts):				
Abscesses	6 (46.15%)	4 (57.14%)	2 (33.33%)	0.246
Nodules	5 (38.46%)	3 (42.85%)	3 (50%)	0.291
Fistulas	2 (15.38%)	0 (0%)	1 (16.66%)	0.823

**Table 3:** Clinical subtypes of HS and distribution between variables.

	Axillary Mammary Phenotype (*N* = 20)	Follicular Phenotype (*N* = 17)	Gluteal Phenotype (*N* = 12)	*p*-Value
Gender				
Female	9 (45%)	7 (41.17%)	5 (41.66%)	0.651
Male	11 (55%)	10 (58.82%)	7 (58.33%)	0.743
Smoking status				
Smoker	12 (60%)	7 (41.17%)	5 (41.66%)	<0.001
Non-smoker	8 (40%)	10 (58.82%)	7 (58.33%)	
Hurley Stage				
I	13 (65%)	6 (35.29%)	6 (50%)	0.231
II	5 (25%)	3 (17.64%)	4 (33.33%)	0.482
III	2 (10%)	8 (47.05%)	2 (16.66%)	<0.001

**Table 4:** Previous treatments for HS patients.

Medication/Treatment	Patients (*n* = 49)	Efficacy (*N*, %)
Topical treatment		
Antiseptics	29 (59.18%)	Partial (*N* = 28, 96.55%) None (*N* = 1, 3.44%)
Antibiotics	46 (92.03%)	Partial (*N* = 44, 95.65%) None (*N* = 1, 2.17%) Complete (*N* = 1, 2.17%)
Intralesional corticosteroids	10 (20.40%)	Partial (*N* = 7, 70%) None (*N* = 2, 20%) Complete (*N* = 1, 10%)
Systemic treatment		
NSAIDs	5 (10.20%)	Partial (*N* = 4, 80%) None (*N* = 1, 20%)
Doxycycline	31 (63.26%)	Partial (*N* = 24, 77.41%) None (*N* = 4, 12.90%) Complete (*N* = 3, 9.6%)
Rifampicin + Clindamycin	17 (34.69%)	Partial (*N* = 15, 88.23%) Complete (*N* = 2, 11.76%)
Clindamycin	10 (20.40%)	Partial (*N* = 9, 90%)) Complete (*N* = 1, 10%))
Penicillin + Clavulanic acid	5 (10.20%)	Partial (*N* = 1, 20%) None (*N* = 3, 60%) Complete (*N* = 1, 20%)
Retinoids	7 (14.28%)	Partial (*N* = 5, 71.42%) None (*N* = 1, 14.28%) Complete (*N* = 1, 14.28%)
Adalimumab	14 (28.57%)	Partial (*N* = 12, 85.71%) Complete (*N* = 2, 14.28%)
Interventions		
Previous incisions and drainage	24 (48.97%)	Partial (*N* = 12, 50%) None (*N* = 2, 8.33%) Complete (*N* = 10, 41.66%)

**Table 5:** Most commonly prescribed treatments for HS patients.

Medication/Treatment	Patients (*n* = 49)
Topical treatment	
Antiseptics	49 (100%)
Clindamycin	44 (89.79%)
Intralesional corticosteroids	1 (2.04%)
Systemic treatment	
Rifampicin + Clindamycin	8 (16.32%)
Doxycycline	17 (34.69%)
Clindamycin	7 (14.28%)
Retinoids	3 (6.12%)
Biologics	
Adalimumab	11 (22.44%)
Secukinumab	5 (10.20%)
Interventions	
Incisions and drainage	3 (6.12%)

## Data Availability

The data that support the findings of this study are available from the corresponding author upon reasonable request.
